# Work-Related Stress, Professional Respect, and Psychological Counseling Among Nurses: A Cross-Sectional Study

**DOI:** 10.1155/jonm/2413658

**Published:** 2025-07-17

**Authors:** Hanif Ullah, Safia Arbab, Sher Alam Khan, Chang-qing Liu, Muhammad Fayaz, Yali Tian, Ka Li

**Affiliations:** ^1^Medicine and Engineering Interdisciplinary Research Laboratory of Nursing & Materials/Nursing Key Laboratory of Sichuan Province, West China Hospital, Sichuan University/West China School of Nursing, Sichuan University, Chengdu 610041, Sichuan, China; ^2^Department of Pediatrics, Musharraf Medical Complex, Abbottabad, Pakistan; ^3^Department of Pediatrics, Abbottabad International Medical Institute, Abbottabad, Pakistan

**Keywords:** mental health, nurses, occupational stress, psychological counseling, respect

## Abstract

**Background:** Occupational stress refers to the psychological pressure from work-related factors. Stress overload is a key contributor to the global nursing shortage. Excessive workloads and psychological pressure further exacerbate stress among healthcare professionals.

**Aims:** This study aims to examine the impact of psychological counseling (PC), job characteristics, and perceived respect on occupational stress among Pakistani nurses.

**Methods:** This cross-sectional study was carried out by nurses from Pakistan, using a random sampling method. Data were collected between April 1 and May 31, 2024. We have access to the nurses' work-related stress, respect, and PC through an online questionnaire. A total of 292 nurses took part in the study.

**Results:** Out of 292 participants, 276 completed the survey, with 71.38% male and 28.62% female. The main causes of occupational stress were low income (95.56%), high workload (80.80%), occupational injury (65.95%), family factors (64.50%), strict leadership (60.50%), and physical problems (56.16%). Additionally, 36.23% of nurses had limited faith in the effectiveness of PC, followed by a moderate belief. According to multiple logistic regression analyses, a correlation between a PC high work-related stress significantly affects nurses' mental health, increasing the need for PC (*p*=0.0979). Stress relief methods like reading, music, or outdoor sports reduce the stress (OR 2.298–11.031, *p* < 0.001). At the same time, factors like nurse–patient relationships and strict leadership contribute to high-stress levels (*p*=0.417, *p*=0.682), with reducing work intensity showing minimal impact (*p*=0.993).

**Conclusion:** Our study indicated that low salaries, a high workload, and low respect could cause occupational stress among nurses who need high PC.

**Implications for Nursing Management:** These findings can guide hospital administrators and nurse managers in enhancing rewards, reducing work hours, and improving job satisfaction. Additionally, better working conditions and training programs can help mitigate occupational stress and support nurses' mental health.

## 1. Introduction

The nursing occupation has been documented as a stressful profession. In the modern environment, stress at work is a significant health issue for nurses. Stress at work may be damaging to a person's mental and physical health, and high levels of stress have been linked to low productivity and high rates of staff truancy. The American Institute of Stress states that up to 80% of work-related injuries and 40% of job turnover are directly related to stress [1]. Many people perceive nursing as a demanding profession with complex requirements. High job demands and a mix of excessive authority and responsibility have been determined to be some of the main causes of occupational stress in nursing personnel [2–4]. A nurse's quality of life may be greatly impacted by occupational stress, which can also lower the standard of care provided. Friendly communication, the application of professional knowledge and abilities, close connections, skilled nursing, and compassion for others are all characteristics of the interpersonal practice known as caring [5].

The main stressors among nurses are certainly a heavy workload, inadequate supervision, and a lack of support from higher authority, as demonstrated by a recent study [6]. Stress has negative effects, like irritability, increased employee turnover, and decreased efficiency [7]. The current study shows that stress can lower one's capacity for work and the quality of care provided. Stress nurses are unable to provide their patients with high-quality care [7]. It has been demonstrated that nurses who earn attractive compensation experience lower stress levels, lower absenteeism from work, and greater inspiration [8]. Additionally, a different study found that high levels of stress can lead to job dissatisfaction; nevertheless, three factors, professional performance, communication autonomy, and appreciation, can improve nurses' job satisfaction [9]. An organization's productivity is greatly influenced by its employees' ability to work in a stress-free environment [10]. A widely accepted recent theory for nurse job-related stress is the Job Demands-Resources (JD-R) Model proposed by Demerouti et al. According to this model, stress develops when the demands of a job, such as high workload, emotional demands, and time pressure, are not balanced by available resources like supervisory support, autonomy, and professional development opportunities. In nursing, persistent high demands without sufficient resources can lead to emotional exhaustion, depersonalization, and ultimately burnout. Recent studies continue to support its relevance in understanding nurse stress and burnout [11].

Stress can negatively impact a nurse's physical and mental health as well as their ability to function professionally [12]. Stress at work has the potential to lower nurses' quality of life, which could lower nursing care quality [13]. The effectiveness of nursing care increases with the quality of life of the nurses [14]. However, stress related to hospital practice affects about one-third of nurses worldwide [15]. Studies showed that over half of nurses experienced stress at work [16]. In a comparable Nigerian survey, around 4/5 of nurses reported moderate job stress, and 10.62% reported severe job stress [17]. According to a survey conducted in Ethiopia, more than half of nurses report experiencing work-related stress, with workload being the most cited stressful subscale among nurses. The likelihood of dying and death comes next [18]. These stressful circumstances may have an impact on nurses' patient safety and cultural practices. Respecting the fundamental worth and dignity of another individual is a moral precept known as respect [19]. Nearly 70% of nurses are satisfied with nurse–physician partnerships, while 47.6% feel respected by patients, and 37.5% feel acknowledged by society [20]. Nurses' intentions to leave are strongly influenced by patient respect, physician–nurse coordination, and job satisfaction [21].

The practice of referring to one's emotional needs and intention to actively seek out psychological and mental health services is known as psychological counseling. Work productivity is severely impacted by poor mental health, and career stability is affected [22]. Effective psychological counseling can assist people in keeping a stable and positive mindset, which helps them deal with unexpected events and lowers their chances of developing mental disorders like depression and anxiety [23]. During devastating infectious disease outbreaks, as the COVID-19 pandemic, psychological counseling has been crucial in helping nurses' mental health [24].

Pakistan is a country with a high population density, inhabited by 197 million people with a low-to medium-level economy. In Pakistan, there were high rates of burnout and fatigue among healthcare workers. In a cross-sectional study on burnout, emotional exhaustion was reported by 42.4% of the 179 healthcare workers in emergency departments [25]. In 2019, a study including 118 surgical residents in Karachi found that women experienced higher levels of emotional fatigue (49.2%) compared to men (50.8%). Married people showed higher levels of personal contentment and sleep scarcity than single people did [26]. During the first wave of COVID-19, 400 healthcare workers were evaluated in six hospitals in Pakistan using the PHQ-9 and GAD-7 scales. The results showed that 21.8% of the workers had moderate-to-severe depression or anxiety [27]. Similar findings were observed in a study including 398 Punjabi healthcare workers, where depression and anxiety were prevalent at 21.4% and 21.9% [28]. In a study conducted during the second COVID-19 wave, 87 healthcare workers reported feeling psychologically exhausted 54% of the time, depersonalized 77% of the time, and low personal accomplishment in 31% of the cases [29]. Pakistani nurses perceive high occupational stress due to heavy workloads, job hazards, and negative interactions, especially in public hospitals [30].

In Pakistan, nurses face significant occupational stress due to high workloads, low monthly salaries, strict leadership, and challenging nurse–patient relationships. Additionally, nurses receive low societal respect and often face criticism from doctors and managers, impacting their mental well-being. Limited access to psychological counseling further exacerbates stress, highlighting the urgent need for better working conditions, fair salaries, and professional recognition to support nurses in the healthcare system. Previous research is limited and lacks clarity on the combined impact of work-related stress, professional respect, and psychological counseling among nurses in Pakistan. This study is critical as it addresses a significant gap in understanding the combined impact of work-related stress, professional respect, and psychological counseling on nurses in Pakistan.

This study falls within the field of occupational health, specifically focusing on Pakistan's nursing profession. It investigates the interplay among work-related stress, perceived professional respect, and the availability of psychological counseling services. The nursing workforce often faces high stress due to demanding work environments, insufficient recognition, and limited mental health support. This study aims to explore the relationship among work-related stress, professional respect, and psychological counseling among nurses in Pakistan. It seeks to assess the prevalence of occupational stress, evaluate perceived professional respect, and examine the availability and use of psychological counseling services.

## 2. Materials

### 2.1. Study Design

The study was conducted at different government and private health institutions in Pakistan. Participants in a questionnaire survey were chosen using a convenience sampling method.

### 2.2. Inclusion and Exclusion Criteria

The study included nurses who voluntarily consented to participate and had at least 1 year of hospital work experience. Exclusion criteria comprised nurses on leave and those still undergoing training. Before conducting the survey, we thoroughly explained the study's objectives and significance to ensure participants fully understood its purpose. Data collection proceeded only after obtaining written informed consent from all participating nurses.

### 2.3. Data Collection

The data were collected from April 1, 2024, to May 31, 2024. A survey was created with Google Forms (https://docs.google.com/forms/), and the link was shared via WhatsApp. All survey questions had to be answered; thus, only completed surveys were included. The nurse assisted in distributing the surveys to individuals and WhatsApp groups. It took 10–15 min to complete the questionnaire [30]. A total of 292 questionnaires were collected, out of which 276 were included in the final data analysis after excluding those that did not meet the criteria. The required sample size was calculated from the Raosoft sample size calculation [31]. The required sample size was determined using the Raosoft sample size calculator, based on a population size of 500, a 50% response distribution, a 5% margin of error, and a 95% confidence interval, resulting in a minimum sample size of 218. To enhance accuracy, 292 responses were collected; however, 16 were excluded due to incomplete or inconsistent data. Two hundred and seventy-six valid questionnaires were included for analysis.

### 2.4. Measures and Instruments

The survey was conducted in English, which is Pakistan's official language. The survey itself consisted of three parts. In the first section, participants addressed 13 questions about their gender, age, marital status, degree of education, place of employment, department, years of experience, job as a nurse, satisfaction with their night shift, and consideration of changing occupations [30, 32]. The second part of the study comprised 23 items on a self-administered questionnaire that assessed the causes of stress (physical, familial, and work-related factors), how respondents decompress (sports, travel, interpersonal relationships), and the degree of respect they felt they received from others, patients, and employees [33]. Part three, which consisted of eight items, assessed the nurses' perceived need for psychological therapy using a Likert scale and explored possible ways to relieve stress, such as better working conditions, more pay, and tremendous respect, among other things. A few sections evaluated the effects of workplace regulations. The responses to these questions were provided using a five-point ordinal scale (1 = none, 2 = a little, 3 = moderately, 4 = strong, 5 = extremely) [33–35].

### 2.5. Statistical Analysis

Respondent data were filled into Google Sheets initially and then transferred to Microsoft Excel and SPSS 22.0. Descriptive and inferential statistics were incorporated in the data analysis, which was predicated on the quantitative analysis. For every study variable, whether dependent or independent, descriptive statistics (univariate analysis) were run using frequency counts or percentages. Multiple multivariate logistic analyses were applied to analyze the various factors related to stress and the need for psychological counseling. The significance level was < 0.05.

### 2.6. Ethical Statement

The Abbottabad International Medical Institute in Abbottabad's Ethical Research Committee has given its approval for the current study (AIMI-Atd/ETH-38-Ped/24). Respondents must assent (accept or deny) to the informed consent declaration on the first page to take part in our online survey.

## 3. Results

### 3.1. Sociodemographic and Work-Related Characteristics of Nurses

A total of 292 online questionnaires were collected from different public and government hospitals, and 276 valid questionnaires were included for final analysis.  Table 1 presents the demographic and professional characteristics of the study participants. Most of the respondents were male, 71.38%, while female participants comprised 28.62%. Regarding marital status, 57.60% of respondents were single, whereas married individuals constituted 42.39%. In terms of educational qualifications, the highest proportion of participants held a Diploma in Nursing (47.10%), followed by those with a Bachelor of Science in Nursing (BSN) and a Master of Science in Nursing (MSN). Employment-type analysis revealed that most participants were employed regularly 64.50%, while 35.50% were under contractual employment. Concerning professional categorization, the largest proportion of participants were general clinical nurses, 60.50%, followed by technicians and head nurses. Departmental distribution indicated that the highest percentage of respondents were from Internal Medicine 28.26%, followed by other departments (22.46%), whereas the lowest representation was from the Intensive Care Unit (ICU) (6.52%).

Concerning work experience, most participants had between 1 and 5 years of experience (70.28%), followed by those with 5–10 years of experience, while 13.76% had ≥ 11 years of experience. Job satisfaction analysis showed that 39.49% of participants were very satisfied with their job, 27.17% were satisfied, 25.36% reported a neutral stance, and 7.97% were dissatisfied. Regarding night shift duties per week, 32.60% of nurses reported having no night shifts, while 26.81% had two-night shifts per week, and 25.36% had three-night shifts per week. Concerning daily working hours, 55% of participants worked 8 h shifts, 31.15% worked 9–12 h shifts, and 13.76% worked 12 h shifts per day, as detailed in Table 1.

### 3.2. Factors of Stress and Characteristics Help Relieve the Stress of a Nurse

According to nurses, the main factors contributing to stress in their profession include heavy workloads, professional responsibilities, job-related risks, and challenging doctor–patient relationships, especially in public hospitals. Many nurses reported that their stress was due to a combination of underlying factors. As shown in Table 2, the leading cause of nurse stress is occupational injury (65.95%), followed by family-related factors (64.50%), strict leadership (60.50%), nurse–patient interactions (59%), and physical health issues (56.16%). A lack of professionalism also contributed to stress for 38.76% of nurses.

To alleviate stress and enhance job satisfaction, nurses identified several methods. The most common stress-relief strategies included maintaining communication with colleagues and family (82.60%), engaging in outdoor activities (80%), going on vocational trips (78.62%), and participating in social gatherings (77.64%). Additionally, the study revealed that 76.45% of nurses felt the need for professional psychological counseling due to the high levels of occupational stress they experience.

Improving psychological counseling services for nurses is essential for their well-being and the quality of care they provide. Nurses believe that reducing stress can be achieved by fostering a respectful and supportive work environment, lowering work-related stress, offering higher pay, and promoting strong interpersonal relationships. Most nurses (95.56%) felt that their salaries were insufficient, contributing to their stress, and 80.80% agreed that reducing work-related intensity was crucial in managing stress, as highlighted in Table 2. By implementing these strategies, hospital administration can create a more supportive environment that enhances nurses' mental health and overall job satisfaction.

### 3.3. Respect Factor of Nurse and PC

Respect plays a fundamental role in the relationship among psychological counseling nurses, their patients, and the broader public. Nurses deserve recognition and respect from patients' families and healthcare teams for their compassion and dedication.

However, findings indicate that 26.1% of nurses reported not receiving adequate respect from patients and their families. Additionally, 18.50% of nurses experienced a lack of respect due to perceived low social status. Furthermore, 37.70% of participants believed they received minimal respect from nurse managers, while 34.05% reported experiencing criticism and insufficient respect from doctors. Regarding psychological counseling support, 36.23% of participants perceived they had little psychological counseling, while others reported moderate or no support. Notably, 7.605% of nurses expressed a strong need for psychological counseling, as detailed in Table 3.

### 3.4. Multiple Logistic Regression Analyses for Respect and Psychological Counseling of Nurses

Different levels of psychological care are required depending on how much society, patients, nurse managers, and physicians respect nurses. This has a significant impact on nurses' mental health, particularly nurses who experience significant levels of work-related stress and who typically report needing psychological counseling more frequently. According to multiple logistic regression analyses, factors influence occupational stress, stress relief methods, societal respect for nurses, ways to reduce stress, and the need for psychological counseling. Physical factors (OR = 1.824, *p*=0.435) and nurse–patient relationships (OR = 0.333, *p*=0.417) contribute to stress, while strict leadership (OR = 1.714, *p*=0.682) also plays a role. Effective stress relief methods include reading or listening to music (OR = 5.035, *p* < 0.001), while other methods like outdoor sports and vacation travel showed no significant impact (*p*=0.999). Nurses face low societal respect, with criticism from doctors and managers having insignificant effects (OR = 0.999, *p*=0.997). Factors like salary improvement, a better work environment, and recognition were seen as potential stress reducers, but their statistical significance was minimal (*p* < 0.001). Reducing work intensity (OR = 6.231E9, *p*=0.993) showed no substantial effect, as shown in Table 4.

## 4. Discussion

Nurses experience varying levels of occupational stress, with those feeling undervalued by society, patients, and fellow healthcare professionals reporting higher stress levels and a greater need for psychological counseling. The majority of nurses agreed that factors such as mutual respect, a supportive work environment, increased compensation, and strong interpersonal relationships could significantly alleviate occupational stress and reduce the demand for psychological counseling [36]. This study is important because it highlights the intense work-related stress nurses face, which can severely impact their mental health, job performance, and patient care quality. By examining professional respect and access to psychological counseling, the research identifies critical gaps in the healthcare system that need urgent attention. It also raises awareness about the need for supportive workplace environments, and hospital administrators design better mental health support services for nurses.

In the present study, most nurses were male, highlighting a significant gender disparity in the nursing profession in Pakistan, where male nurses outnumber their female counterparts. This imbalance may be attributed to higher educational attainment among males compared to that among females; the results obtained were similar to those of previous reports [37, 38]. Compared to reports from India and European nations, the percentage of female nurses was significantly greater [39, 40]. Pakistan's healthcare workforce has a notable gender imbalance, with men outnumbering women due to societal barriers and limited educational access. Addressing these challenges requires inclusive policies to promote gender equality in healthcare professions.

In the current study, 60.50% of nurses were aged between 18 and 30 years, while only 13.76% had more than 10 years of job experience. The study also found that most of the nursing personnel working in the clinical setting were inexperienced and young. This trend raises concerns about the potential challenges younger nurses may face when dealing with high-pressure environments and complex patient care situations. Additionally, it was observed that 31.15% of nurses were required to work approximately 8–12 h per day, while 55% of nurses were required to work 8 h per day. In Pakistan, healthcare workers typically work around 48–56 h per week, often exceeding this due to staff shortages and emergency demands. Long and irregular shifts, including night duties, are especially prevalent in public sector hospitals, potentially leading to increased work-related stress, burnout, and compromised patient safety. In Saudi Arabia, the standard working hours for healthcare workers are generally 8 h per day, totaling 48 h per week, as specified by the Ministry of Human Resources and Social Development [41]. Similarly, the People's Republic of China's “Labor Law,” item 36, states that all workers from various industries should not work more than 8 hours a day [22]. Considering the responsibilities assigned to each nurse, the study found that nurses typically worked around 56 h per week, exceeding the standard 55 h workweek, leading to increased work intensity. Excessive working hours contributed to greater pressure from employers, potentially resulting in job burnout. Additionally, longer shifts were significantly correlated with burnout and a higher intention to quit [37, 38].

Nurses in Pakistan face high workloads, extended workdays, and adverse conditions, leading to occupational injuries and job-related challenges. In this study, 65.95% of nurses developed work-related disorders and injuries, with low income 95.56% further contributing to stress and dissatisfaction. These factors negatively impact their quality of life and professional growth, highlighting the need for better compensation and improved working conditions to enhance retention and well-being. The study reported that 67.7% of participants identified occupational injuries as a major contributor to their stress levels, while 43.8% attributed their stress to physical health problems [35]. These findings support the idea that both professional and personal factors significantly impact the psychological well-being of nurses, reinforcing the urgent need for comprehensive support systems addressing both workplace safety and personal life stressors. These outcomes aligned with the findings of the previous research [42]. Studies in the past have indicated that jobs requiring extended standing during working hours may put workers at risk for muscle problems. One of the physical demands of the nursing profession is standing for extended periods. Another is moving patients from beds to stretchers, which requires physical activity that can cause harm to the individual's health [43, 44].

The study found that 64.50% of nurses experienced stress from non-work-related factors, such as family issues, which significantly contributed to their overall stress levels. Similarly, Zhang et al. reported that 70.8% of nurses identified family-related stress as a major source of their psychological burden. These findings suggest that addressing both professional and personal stressors is essential for improving nurses' mental well-being [35]. In the present study, 65.85% of nurses reported experiencing a high workload; Zhang et al. found that 58% of nurses faced high workload pressures. This comparison highlights the particularly demanding conditions faced by nurses in the current study setting. The results of the study were supported by another study indicating that an excessive workload causes significant stress [45]. Additionally, a study carried out in Jimma Zone, South West Ethiopia, revealed that a workload of 57.6% in public health hospitals was noticeably higher and lower than our study [7]. Another study revealed that a significant cause of stress was workload, and it was carried out at two specialized hospitals in the city of southwest Nigeria and an Italian healthcare company [46, 47]. Similarly, Faremi et al. observed that among the commonly reported stressful occurrences were an excessive number of non-nursing jobs, such as administrative labor, an insufficient number of staff members to meet the ward burden, an absence of drugs and equipment needed for nursing care, and unpredictable staffing and scheduling [46]. A lack of nurses, poor assistance from nursing supervisors, low pay, and excessive expectations were also mentioned as administrative problems [48]. Because of the longer patient stays, lower staffing, greater overtime, and increasing demand for nurses, nurses are working more than they have in the past [49].

According to the current study, nurses who worked longer shifts at the hospital every week were likely to experience a variety of professional stressors. The data analysis results showed that the incidence and kind of occupational diseases among nurses were substantially correlated with years of work experience, working nights, and the number of hours worked per week. It is important to note that compared to other professions, nurses have an excessively high workload and long work hours [44, 50]. Overwork will lead to bodily harm, emotional distress, extended working hours, and ultimately the emergence of certain occupational diseases. Numerous nurses reported having these observations [51, 52]. Consequently, the prevalence of work-related stress might be higher than previously considered. Numerous occupational disorders can result in a deterioration of physical abilities and a reduction in productivity, which can have an impact on nurses' career planning, job satisfaction, and personal growth.

Occupational diseases are a concern in nursing due to constant exposure to a variety of physical, chemical, biological, and psychosocial risks [53]. Nurses commonly experience musculoskeletal disorders from patient handling, infectious diseases due to contact with patients' bodily fluids, and mental health problems such as stress and burnout caused by heavy workloads and emotional strain [54]. In particular, the psychological burden of continuous patient care and exposure to suffering contributes significantly to anxiety, depression, and sleep disturbances among nurses. Therefore, improving workplace conditions and preventive measures is critical to reducing occupational diseases and promote nurses' well-being.

In the present study, 60.50% of nurses reported experiencing stress due to strict leadership. This finding is consistent with international studies, such as those by Letvak et al. who found that authoritarian leadership styles contribute significantly to work-related stress in nurses. The study suggests that supportive leadership styles lead to lower stress levels and better nurse retention. This highlights the need for more flexible and empowering leadership approaches to mitigate stress in the nursing profession [54]. Babapour et al. findings indicate that job stress has a moderate negative relationship with psychological well-being and a weaker effect on physical health, with 27.9% of the variance in quality of life explained by job stress [13]. This is consistent with the work of Ervasti et al. who found that job stress significantly impacts mental health, but with varying degrees of effect on physical health [55]. However, studies like Sonnentag et al. have shown stronger associations between job stress and both psychological and physical outcomes, suggesting that contextual factors may influence these relationships [56].

Respect is important for providing the best possible care for patients and is frequently seen as a behavior that aids in a person's recognition of their value and worth, which in turn affects their attitudes and sentiments [57]. Our study found that nurses received little respect from patients and their families. About 32.24% stated that adequate respect was essential in reducing occupational stress and the need for psychological counseling. Few studies have investigated the respect nurses receive from people they assist, even though several have examined the regard patients receive from nurses. Although it is challenging to quantify views of respect precisely, a lot of nurses reported high levels of professional pressure and overwork, which may have distorted their view of the respect they may have gained at work. Specifically, addressing the criticism that nurses perceived from strict leadership and high workload, multiple logistic regression analyses demonstrated a correlation between a higher need for psychological counseling and lesser respect perceived by nurses. Therefore, appreciating and valuing nurses could increase their intrinsic motivation, which would strengthen their professional motivation and values [58]. Persistent nurse respect has also been linked to improved autonomy and decision-making skills for nurses as well as increased communication between doctors and nurses, all of which may have a significant influence on the course of a nurse's career [59]. This type of counseling often makes use of a variety of communication techniques to reduce stress by altering a person's perception of themselves, enhancing their capacity for behavior, and offering direction for personal growth [60, 61]. Notably, all study participants indicated a need for psychological counseling, which is more than previously documented and may suggest that psychological issues that follow professional stress in nurses are more significant than previously thought [62].

### 4.1. Limitations of the Work

This study has several limitations, even if it improves through empirical opinion in the discussion about the nurses. First, self-selection bias might have had a role because the study was conducted online. It is not possible to extrapolate the results to other circumstances because a convenience sample was utilized, and the results might not accurately represent the population. Because the study's design was cross-sectional and it assessed occupational stress at a certain point in time, care must be taken when interpreting the findings. Despite our finding that several factors affect nurses' mental health, it is still unknown what mediates the relationship between these factors and the necessity of psychological counseling. Management guidelines proved unproductive due to public insignificance. Particularly, there are more private hospitals in Pakistan than state-run ones, and their workforce is composed of more medical professionals and doctors in particular than nurses and other healthcare workers [63]. Furthermore, there is a notable disparity in gender among nurses, with a larger number of males than females. There is a notable gender imbalance in Pakistan's healthcare workforce, with men outnumbering women in many areas. Pakistan's healthcare workforce faces a significant gender imbalance, with men outnumbering women in many sectors. This disparity stems from persistent challenges in women's empowerment and limited access to education. Cultural norms and societal barriers often restrict opportunities for women, particularly in healthcare professions like nursing and nursing assistance, where male workers dominate. Despite efforts to promote gender equality, these challenges persist, emphasizing the need for more inclusive policies. The lower rate of female education is largely due to societal preferences that prioritize male education over female learning opportunities. These trends highlight the need for more inclusive policies to encourage women's participation in these essential roles [30]. A longitudinal study that examines developments in the mental health symptoms evaluated in this research over time would be an excellent approach to address this.

## 5. Implications for Nursing Management

The fundamental component necessary to sustain regular nursing work was the staff members' health. Nursing managers must actively implement several programs, including health assessments, training, and prevention plans, among nurses to lower the incidence of occupational diseases. Programs for peer social support and stress management are important for lowering occupational stress and should be successful interventions in improving nurses' job satisfaction. Governments must take the necessary actions to safeguard the mental and physical health that form the cornerstone of the healthcare system. It is recommended that governments implement financial subsidies and incentive schemes to raise the level of job satisfaction among nurses. Our findings suggest that by creating structural empowerment strategies, hospital administration and nurses can improve the psychological empowerment of nurses. Furthermore, we advocate for the development of services, programs, and counseling aimed at promoting the mental well-being of healthcare professionals. Nurse managers must also offer opportunities for career growth and vocational training to their staff, in addition to continuing education programs and workplace assistance, as these factors may have an impact on the nurses' job satisfaction, intention to leave, and career advancement.

## 6. Conclusion

In conclusion, this study highlights the significant impact of occupational stress on the mental health of nurses in Pakistan, particularly in terms of stress. Our findings underscore that workload, family factors, and low pay are key contributors to job-related stress, affecting nurses across various domains of their lives. However, the research also reveals that improving working conditions, increasing pay, fostering strong interpersonal relationships, and ensuring respect in the workplace can effectively alleviate stress and reduce the need for psychological counseling. These factors are crucial in enhancing nurses' well-being, job satisfaction, and overall quality of life. To address the challenges identified, healthcare institutions should focus on creating supportive work environments and promoting mutual respect among healthcare professionals.

## Figures and Tables

**Table 1:** Demographic and work-related characteristics of 276 nurses.

Variable	*N* (%)
*Gender*	
Male	197 (71.38)
Female	79 (28.62)

*Marital status*	
Single	159 (57.60)
Married	117 (42.39)

*Age*	
18–30	167 (60.50)
31–40	51 (18.47)
> 41	58 (21)

*Education*	
BSN	114 (41.30)
Diploma in nursing	130 (47.10)
MSN	17 (6.15)
Post-RN BSN	15 (5.43)

*Professional title*	
General clinical nurse	167 (60.50)
Head nurse	28 (10.14)
Technician	81 (29.34)

*Monthly salary*	
50,000 PKR	132 (47.82)
> 60,000 PKR	90 (32.60)
50,000–60,000 PKR	54 (19.56)

*Type of employment*	
Contractual	98 (35.50)
Regular	178 (64.50)

*Work experience (years)*	
> 11	38 (13.76)
1–5	194 (70.28)
5–10	44 (15.94)

*Working hours per day (h)*	
8	152 (55)
> 12	38 (13.76)
9–12	86 (31.15)

*Workload is very heavy*	
No	51 (18.47)
Normal	96 (25)
Yes	129 (46.73)

*Department*	
Emergency dept	58 (21)
ICU	18 (6.52)
Infectious disease ward	34 (12.31)
Internal medicine	78 (28.26)
Others	62 (22.46)
Surgical	26 (9.42)

*Job satisfaction*	
General	70 (25.36)
Not satisfied	22 (7.97)
Satisfaction	75 (27.17)
Very satisfied	109 (39.49)
*Number of night shifts per week*	
0	90 (32.60)
1	39 (14.13)
2	74 (26.81)
≥ 3	70 (25.36)

**Table 2:** Factors of stress and characteristics help relieve the stress of nurse.

Variable	*N* (%)
*Factor of stress*	
Family factors	
No	98 (35.50)
Yes	178 (64.50)
Nurse–patient relationship	
No	113 (41)
Yes	163 (59)
Physical factors	
No	121 (43.84)
Yes	155 (56.16)
Lack of professionalism	
No	169 (61.23)
Yes	107 (38.76)
High workload from hospitals	
No	97 (35.15)
Yes	179 (65.85)
Strict leadership	
No	109 (39.50)
Yes	167 (60.50)
Occupational injury	
Yes	182 (65.95)
No	94 (34.05)

*Factors to relieve stress of nurses*	
Vacation travel	
No	59 (21.38)
Yes	217 (78.62)
Outdoor sports	
No	55 (20)
Yes	221 (80)
Chatting with family and friends	
No	69 (25)
Yes	207 (75)
Chat with colleagues	
No	48 (17.40)
Yes	228 (82.60)
Friendly working environment	
No	13 (4.71)
Yes	263 (95.29)
Getting respect	
No	10 (3.62)
Yes	266 (96.37)
Realizing the value of your life	
No	19 (6.88)
Yes	257 (93.12)
PC with professionals	
No	65 (23.55)
Yes	211 (76.45)
Participating in parties	
No	62 (22.46)
Yes	214 (77.64)
Promotion of occupation	
No	15 (5.50)
Yes	261 (94.50)
Listening or reading to music	
No	145 (52.54)
Yes	131 (47.46)
Improving salary level	
No	12 (4.34)
Yes	264 (95.65)
Reducing the work-related intensity	
No	53 (19.20)
Yes	223 (80.80)

**Table 3:** Respect factor of nurse and PC.

Respect factor	None%	Little%	Moderately%	Strong	Seriously
Criticism to nurses from doctors	63 (22.82)	94 (34.05)	58 (21)	39 (14.13)	22 (8)
Respect from patients and their families	73 (26.44)	89 (32.24)	47 (17)	41 (14.85)	26 (9.45)
Criticism to nurses from nursing managers	72 (26.1)	104 (37.70)	47 (17)	36 (13)	17 (6.15)
Low social status	51 (18.50)	93 (33.70)	71 (25.72)	51 (18.50)	10 (3.62)
Do you need psychological counseling	77 (27.90)	100 (36.23)	63 (28.82)	21 (7.60)	15 (5.45)

**Table 4:** Using multivariate logistic regression analyses for respect and PC need of Pakistani nurses (*N* = 276).

Variable	SE	Wald	OR (95% CI)	*p* value
*Occupational stress*				
Physical factors	0.769	0.611	1.824 (0.404–8.229)	0.435
Family factors	40,191.69	< 0.001	0.001 (0.001–0.001)	1.000
Nurse–patient relationship	1.354	0.658	0.333 (0.023–4.736)	0.417
Lack of professionalism	0.365	0.046	1.082 (0.529–2.210)	0.830
High work requirements from hospitals or departments	40,191.69	< 0.001	7.588E8 (0.001–0.001)	1.000
Strict leadership	1.314	0.168	1.714 (0.131–22.513)	0.682

*Ways to relieve stress*				
Outdoor sports	20,096.59	< 0.001	1.212E9 (0.001–0.001)	0.999
Vacation travel	20,096.59	< 0.001	0.001 (0.001–0.001)	0.999
Chatting with friends and family	20,096.45	< 0.001	0.001 (0.001–0.001)	0.999
Chat with colleagues	0.823	0.243	0.667 (0.133–3.344)	0.622
Reading or listening to music	0.400	16.316	5.035 (2.298–11.031)	< 0.001
Psychological counseling with professionals	20,096.45	< 0.001	8.077E8 (0.001–0.001)	0.999
Participating in parties	1.732	0.641	4.000 (0.134–119.230)	0.423

*Respect to nurses*				
Low societal status	7.307E3	< 0.001	0.001 (0.001–0.001)	0.999
Difficult to get respect of patients and families	1.210E4	< 0.001	0.001 (0.001–0.001)	0.999
Criticize to nurses from doctors	1.803E4	< 0.001	0.001 (0.001–0.001)	1.000
Criticize to nurses from nursing managers	1.156E4	< 0.001	1.913E18 (0.001–0.001)	0.997
Do you need psychological counseling?	2.790E3	< 0.001	9.153E31 (0.001–0.001)	0.979

*What factors could be improved to relieve your stress and improve the necessity for psychological counseling?*				
Good working environment	4.923E4	< 0.001	1.000 (0.001–0.001)	1.000
Improving salary level	4.923E4	< 0.001	1.000 (0.001–0.001)	1.000
Getting respect	3.113E4	< 0.001	1.000 (0.001–0.001)	1.000
Promotion of occupation	3.481E4	< 0.001	1.000 (0.001–0.001)	1.000
Reducing the work-related intensity	2.692E3	< 0.001	6.231E9 (0.001–0.001)	0.993
Realizing the value of your life	2.010E4	< 0.001	4.188E8 (0.001–0.001)	0.999

## Data Availability

The data that support the findings of this study are available on request from the corresponding authors. The data are not publicly available due to privacy or ethical restrictions.
